# Advanced Diffusion MRI in Cervical Cancer: A Comprehensive Review

**DOI:** 10.3390/diagnostics16121870

**Published:** 2026-06-16

**Authors:** Ali S. Alyami

**Affiliations:** Diagnostic Radiography Technology (DRT) Department, College of Nursing and Health Sciences, Jazan University, Jazan 45142, Saudi Arabia; aalmansour@jazanu.edu.sa

**Keywords:** cervical cancer, IVIM-DWI, DTI, treatment response monitoring, precision oncology, parametrial invasion

## Abstract

Advanced diffusion MRI techniques, particularly intravoxel incoherent motion (IVIM) and diffusion tensor imaging (DTI), have emerged as promising functional imaging tools for improving cervical cancer assessment beyond conventional anatomical MRI. This narrative review summarizes current evidence on the clinical utility of these diffusion-based techniques for tumor characterization, local staging, parametrial invasion, lymph node evaluation, treatment response monitoring, and emerging radiomics applications. Across studies, diffusion-related parameters, especially the apparent diffusion coefficient (ADC) and pure molecular diffusion coefficient (D), tend to be lower in malignant cervical tissues and correlate with increased cellularity, higher tumor grade, and more aggressive disease features. IVIM metrics appear especially useful for differentiating cervical cancer from normal tissue, predicting pelvic lymph node involvement, and detecting early treatment response to chemoradiotherapy or neoadjuvant chemotherapy before substantial morphological regression occurs. In contrast, DTI remains less extensively investigated; however, preliminary findings suggest potential value for evaluating parametrial invasion, stromal disruption, tumor grade, and lymph node metastasis, particularly when integrated with IVIM-derived indices. Although diffusion-derived radiomics may further support risk stratification and treatment-response prediction, the evidence base remains limited by small cohorts, single-center designs, methodological heterogeneity, and insufficient external validation. Overall, IVIM and DTI provide valuable non-invasive insight into cervical cancer biology, but standardized acquisition protocols, reproducible thresholds, and multicenter validation are needed before routine clinical implementation.

## 1. Introduction

Globally, cervical cancer is a significant oncological challenge, ranking as the fourth most prevalent malignancy and the fourth leading cause of cancer-related mortality among women worldwide. Epidemiological data from 2022 indicate a substantial disease burden, with approximately 660,000 new diagnoses and 350,000 deaths worldwide [1]. Persistent infection with high-risk strains of human papillomavirus (HPV) is established as the principal causative factor in the development of cervical carcinoma. Beyond its well-defined role in cervical oncogenesis, evidence confirms the involvement of HPV in the pathogenesis of a broader spectrum of malignancies. Epidemiologically, HPV infection remains the most common sexually transmitted infection globally. The most frequent histological type of cervical cancer is squamous cell carcinoma (70–80%), whereas the glandular type (cervical adenocarcinoma and other epithelial tumors, undifferentiated carcinoma, neuroendocrine tumors, and adenosquamous carcinoma) is less common (20–25%) [2].

Treatment options for cervical cancer include chemotherapy, radiotherapy, and surgery. Decision-making in management primarily relies on staging, which can be determined using the lymph node metastasis or International Federation of Gynecology and Obstetrics (FIGO) 2018 classifications. The determinants of FIGO 2018 staging include the depth of stromal invasion, parametrial extension, tumor size, involvement of the vaginal and pelvic side walls, associated lymphatic spread, renal complications, and involvement of the bladder and/or rectum, as well as metastasis to solid organs [3].

Magnetic resonance imaging (MRI) is a non-invasive technique that is used to diagnose, stage, plan treatment for, and assess response to cervical cancer [4]. However, conventional MRI primarily assesses changes in tumor size, signal intensity and morphology. In contrast, advanced MRI techniques provide more detailed information by assessing the tumor’s biological characteristics to predict treatment outcomes. These techniques, such as intravoxel incoherent motion (IVIM) and diffusion tensor imaging (DTI), rely on diffusion-weighted imaging (DWI) to provide valuable insights into tissue characterization by quantifying both perfusion and diffusion properties. DW-MRI generates contrast by quantifying the random microscopic movement of water molecules. It is well recognized that the ADC values for benign lesions or normal tissues are typically higher than those associated with malignant tumors [5]. Moreover, effective cancer treatment can be indicated by elevated ADC values, which result from changes in tumor cellularity and in cell membrane integrity due to apoptosis and necrosis [6].

IVIM-DWI employs a biexponential model that uses multiple b-values to derive diffusion- and perfusion-related properties of water molecules, thereby providing insights into their movement within tumor tissues without a contrast medium. DTI is an advanced DWI-based technique used to estimate the directionality and strength of water diffusion in different tissue structures. These methods may serve as valuable adjuncts in the evaluation of cervical carcinoma, as they extend characterization beyond morphological characteristics, such as size and local invasion, to provide insight into the tumor’s pathophysiological microenvironment [7,8]. IVIM offers critical insights into cellular diffusion and tumor perfusion in cervical cancer studies, which are essential for understanding angiogenesis and tumor biology. For example, cervical cancer had lower diffusion and perfusion IVIM values compared to leiomyoma tissue and normal cervical tissue. Zhou et al. found higher perfusion at the tumor edge in high-grade tumors and found that D was significantly lower in G3 tumors than in G1 tumors [8]. Despite the growing interest in advanced diffusion MRI techniques, their clinical value in cervical cancer remains uncertain due to inconsistent methodologies and the limited availability of high-quality evidence.

Advanced diffusion MRI techniques, including IVIM and DTI, have demonstrated established clinical utility across multiple malignancies, including prostate [9], breast, liver, and renal cancers, as well as hepatocellular carcinoma, particularly in lesion characterization and treatment response assessment, where these techniques correlate with tumor perfusion, microvascular density, and treatment response, supporting their biological validity. For example, in breast cancer, DTI biomarkers from breast tumors are not only linked to prognostic factors such as lymphovascular invasion and Ki-67 expression levels [10,11] but also show correlations with treatment response [12,13] as well as distinguishing between benign and malignant lesions. Similarly, in hepatic tumors, IVIM-derived parameters such as f and Dfast can differentiate malignancies with distinct pathological characteristics, including distinguishing intrahepatic cholangiocarcinoma from hepatocellular carcinoma [14,15]. Furthermore, in head and neck cancers, IVIM-derived parameters have been shown to differentiate tumor grade and predict treatment response [16]. These applications support the biological validity of diffusion-based imaging biomarkers, although their translation into cervical cancer remains limited due to a lack of standardization and methodological heterogeneity.

Accordingly, this review aims to evaluate the current clinical utility of IVIM and DTI in cervical cancer, assess the reliability of diffusion and perfusion parameters reported across different studies, and identify the key limitations that hinder their translation into routine clinical practice.

## 2. Basic Principles of DWI Types

### 2.1. Intravoxel Incoherent Motion (IVIM)

The fundamental principle of DWI is the measurement of Brownian motion—the random translational displacement of individual water protons. This molecular motion can be quantified by a diffusion coefficient, D. Initially, the signal attenuation resulting from this random displacement induced by diffusion-sensitizing magnetic gradients was modeled with a monoexponential function:

S/S0 = exp (−b × D)

where S measures diffusion weighted signal intensity and S0 represents the corresponding signal intensity in the absence of diffusion weighting. The D (mm^2^/s) parameter characterizes the diffusion coefficient of water, while the b-value (s/mm^2^) is calculated as follows: γ is the gyromagnetic ratio (MHz/T), G is the amplitude of the two diffusion gradient pulses (mT/m), δ is the duration of the pulses (ms), and Δ is the time interval between the two diffusion gradients (ms). IVIM is derived from conventional DWI. The IVIM model, first proposed by Le Bihan et al. in the late 1980s, accounts for translational movements at the voxel level that are not solely due to molecular diffusion [17]. Specifically, it posits that the microcirculation of blood within the capillary network mimics a pseudo-diffusion process.

Sb/S0 = (1 − f) × exp (−b × D) + f × exp (−b × D*)

where D (also termed D_slow) represents pure molecular diffusion, also known as the true diffusion coefficient, representing the slow component of diffusion. Dfast (also called D*) represents incoherent microcapillary perfusion, also known as the pseudo-diffusion coefficient, which denotes the fast component of diffusion. PF (also called f) is the fraction of the fast diffusion compartment, and (1 − PF) is the fraction of the slow ADC. D is sometimes referred to as ADCslow, while D* may also be known as ADCfast.

The IVIM sequence comprises multiple acquisitions initially performed without diffusion gradients, followed by acquisitions using varying gradient amplitudes and durations, corresponding to different b-values. A crucial aspect of the IVIM acquisition protocol is the selection of b-values, specifically regarding their quantity and distribution, which leads to varying degrees of diffusion weighting in the obtained images.

IVIM parameters can be derived using either segmented (two-step) or simultaneous (three-parameter) nonlinear fitting approaches. Segmented methods first estimate D from high b-values (>200 s/mm^2^) and then calculate perfusion parameters from low b-values, while simultaneous methods fit all parameters together. These methodological choices significantly influence parameter stability and reproducibility.

### 2.2. Diffusion Tensor Imaging (DTI)

DTI represents a significant advance over conventional MR sequences, including T1- and T2-weighted imaging, by providing superior visualization of tissue microstructures. As shown by Basser et al., DTI is based on measuring the anisotropic diffusion of extracellular water molecules in white matter [18].

DTI characterizes the directional behavior of water diffusion within tissue microstructure, enabling quantitative assessment of diffusion anisotropy gradients along at least six distinct spatial orientations. This technique maps the three-dimensional mobility of water molecules and identifies any preferential directionality within tissue microstructures. Such directional data enable tractography, a method that generates three-dimensional reconstructions of white matter pathways by tracing the principal diffusion axis within each voxel of the image. Consequently, DTI provides quantitative metrics that characterize both the magnitude and the spatial orientation of water diffusion. This non-invasive technique evaluates diffusion anisotropy by applying magnetic field gradients along multiple spatial directions. These gradients are crucial for assessing the diffusion anisotropy of water molecules within axonal bundles [19]. The diffusion data obtained are then used to create three-dimensional images of white matter pathways in the spinal cord and brain. This process employs specialized fiber-tracking algorithms—for example, probabilistic or deterministic tractography. While these visualized pathways, known as tractography, display the architectural orientation of tissue structures and are often interpreted as individual axons or nerve fibers, it is essential to understand their physical meaning: they represent lines indicating the path of maximum water diffusivity within a voxel. DTI data is mathematically modeled using a diffusion tensor, represented as a symmetric matrix. This tensor describes the three-dimensional shape of water diffusion within a voxel, which is characterized by three principal eigenvalues and their corresponding eigenvectors.

Several quantitative parameters can be acquired from DTI that are useful for evaluating conditions affecting white matter. Fractional anisotropy (FA) quantifies the degree of directional preference of water diffusion within a voxel, ranging from 0 to 1, and serves as a biomarker of axonal integrity, typically decreasing in the presence of white matter pathologies. FA values range from 0 to 1. High FA (approaching 1) indicates highly restricted diffusion in one primary direction, characteristic of highly organized white matter tracts (e.g., intact axons, parallel and tightly myelinated). Low FA (close to 0) suggests isotropic diffusion, meaning water moves equally in all directions, typical of unorganized structures such as cerebrospinal fluid (CSF), gray matter, or diseased or damaged white matter where structural barriers are compromised. Mean diffusivity (MD) provides a more precise measurement than the ADC because it accounts for the three primary axes of water movement. Like FA, MD can be interpreted as high or low. High MD indicates unrestricted water movement (e.g., in areas of severe edema or CSF), whereas low MD indicates highly restricted water movement (e.g., in acute ischemic stroke due to cellular swelling). Axial diffusivity (AD) assesses water diffusion along the principal longitudinal axis, thereby evaluating axonal integrity. In contrast, radial diffusivity (RD) reflects myelin integrity, with myelin damage resulting in elevated RD values.

While DTI was originally developed for neuroimaging, its application in tumors such as cervical cancer leverages sensitivity to tissue architecture, including collagen organization in the stroma and cellular packing density. In this context, FA reflects microstructural integrity rather than white matter tract organization.

## 3. Methods

### 3.1. Search Strategy and Selection Criteria

This study was designed as a narrative review to synthesize current evidence regarding IVIM and DTI diffusion MRI techniques in cervical cancer. Although a structured literature search was conducted, the methodology does not fully adhere to PRISMA guidelines for systematic reviews; therefore, the findings should be interpreted as a qualitative narrative synthesis rather than a quantitative systematic evaluation. This study was designed as a narrative review to synthesize current evidence on advanced diffusion MRI techniques in cervical cancer. Although a structured literature search was undertaken, the methodology does not fully adhere to PRISMA guidelines for systematic reviews; therefore, the findings should be interpreted as a qualitative synthesis rather than a quantitative systematic evaluation.

A comprehensive literature search was performed in December 2025 using the Ovid platform to access the Medline and Embase databases. In addition, Google Scholar was searched to identify potentially relevant studies examining the role and clinical utility of IVIM and DTI in cervical cancer. The search strategy incorporated the following keywords: ‘diffusion tensor imaging’ OR ‘diffusion tensor tractography’, ‘intravoxel incoherent motion’ OR ‘IVIM’, AND ‘cervical neoplasm’, ‘cervical cancer’, ‘cervix carcinoma’, ‘uterine cervical cancer’, ‘cervix cancer’, ‘cancer of the cervix’, or ‘uterine cervix’. Studies were initially screened based on titles and abstracts, followed by full-text assessment of potentially eligible articles. The literature search covered publications from 2015 to 2025.

### 3.2. Selection Criteria

#### Inclusion/Exclusion Criteria

Inclusion criteria required studies involving adult patients (aged ≥ 18 years) with a histopathologically confirmed diagnosis of cervical cancer. Eligible papers were required to utilize DTI and/or IVIM techniques, involve human participants, and be published as full-text articles in the English language. Moreover, studies published between 2015 and 2025 focusing on IVIM and DTI in human subjects with biopsy-proven cervical cancer were included. Exclusion criteria included studies that did not use DTI or IVIM, involved pediatric patients (aged <18 years), were published in non-English languages, or were classified as secondary research (e.g., narrative reviews, meta-analyses, systematic reviews) or conference abstracts. Moreover, studies about combined imaging techniques such as MRI-CT or MRI-PET were excluded.

## 4. Application of IVIM in Cervical Cancer

Several studies have investigated the diagnostic and prognostic applications of IVIM in cervical cancer (Table 1). This is achieved by modeling the signal attenuation caused by the microscopic, pseudorandom motion of water molecules as they diffuse within the tissue parenchyma and flow within the capillary network [17]. A prospective single-center study aimed to assess empirical DW-MRI models in cervical tumors to investigate whether fitted parameters could distinguish between grades and types of tumors [20]. Monoexponential diffusion metrics (e.g., ADC) and IVIM-derived parameters (including the D) were evaluated in 42 cervical cancer patients. Both ADC and D were lower in poorly differentiated tumors than in well or moderately differentiated tumors.

Wu et al. [21] conducted a prospective study to evaluate the dependence of diffusion parameters on selected b-values in IVIM. It also examined the clinical use of multiple diffusion parameters derived from monoexponential (ADC or Dst) and biexponential models (D, D*, f) to differentiate normal cervical from cervical cancer. The study included 120 women with pathologically confirmed cervical cancer and 21 healthy participants with a normal cervix, totaling 141 participants. They found that all tested parameters, except D* for both b-value ranges, significantly differed between cervical cancer patients and controls (*p* < 0.01). Furthermore, ADC 2000, D2000, ADC 1000, and D1000 were significantly higher in the normal cervix group than in the cancer group (*p* < 0.05). D2000 showed the best ability to distinguish between cervical cancer and a normal cervix, with an AUC of 0.923. The AUCs of 0.909 and 0.907 also indicated comparable diagnostic value. D* and ADC 2000 tended to perform better than D1000 in diagnostic efficiency, though the differences were not statistically significant. Additionally, they reported that the overall diagnostic performance of ADC, D, D*, and f was better over the b-value range of 0 to 2000 s/mm^2^ compared to the 0 to 1000 s/mm^2^ range.

Wang et al. [22] conducted a prospective study that compared the reliability of various fitting methods for IVIM parameters (D, f and D*) and assessed their impact on differentiating cervical cancer from normal tissue. The study included 30 participants (20 patients with histologically confirmed cervical cancer and 10 subjects with negative biopsies considered normal tissue). The researcher compared a simultaneous (three-parameter) fitting method against two variations in the segmented (two-step) fitting method, utilizing cut-off b-values of 100 s/mm^2^ (M1) and 200 s/mm^2^ (M2). The study found that no significant difference was found between IVIM parameters derived from the segmented method with a b-value cut-off of 200 s/mm^2^ and the simultaneous fitting method (*p*  >  0.05). The D* value did not show a significant difference between cervical cancer and normal tissue (*p* > 0.5), while both D and f were significantly reduced in cervical cancer compared to normal tissue (*p* < 0.05) across all fitting methods.

Another prospective study aimed at evaluating the feasibility of a parameter-free IVIM imaging algorithm in assessing cervical cancer. The study enrolled 19 female patients with biopsy-proven cervical cancer, classifying them into two histological groups: squamous cell carcinoma (*n* = 10) and adenocarcinoma (*n* = 9) [23]. They reported the feasibility of parameter-free IVIM analysis and reported significant differences in perfusion-related parameters between adenocarcinoma and squamous cell carcinoma.

Perucho et al. [24] conducted a retrospective study examining the utility of IVIM parameters derived from the primary tumor for classifying pelvic lymph node (PLN) involvement in cervical cancer patients. The mean values of D, ADC, and f were all significantly different across the three defined groups of PLN involvement (no metastasis, sub-centimeter metastasis, and metastasis of size significant). The study concluded that combining IVIM-derived diffusion metrics with diffusion tumor volume improved the prediction of pelvic lymph node metastasis and outperformed conventional size-based MRI criteria.

Zhang et al. [25] conducted a prospective single-center study to identify the most effective mathematical model for predicting responses to concurrent chemoradiotherapy in cervical cancer. The study compared three diffusion models: the Gaussian diffusion (monoexponential), IVIM, and stretched exponential models. A total of 84 patients with biopsy-confirmed advanced cervical cancer (FIGO stages IIB–IVA) scheduled to undergo concurrent chemoradiotherapy were included. The authors found that parameters derived from the stretched exponential model showed better predictive performance for treatment response than conventional IVIM parameters.

Lin et al. [26] (2021) conducted a prospective study to investigate digital PMI in 90 patients using DTI and IVIM models. They found that IVIM and DTI parameters in PMI (FA-DTI and D-IVIM) were significantly lower in cases of PMI (FA-0.07 versus 0.09 and D-0.63 versus 0.77). Combining DTI and IVIM produced the best differentiation results (improving the AUC in the receiver operating characteristic curve for D (0.80) and FA (0.73) to 0.93).

Song et al. [27] conducted a prospective study using various models derived from DWI, including monoexponential/standard-exponential models (MEM/SEM), IVIM with lower b-values, and DKI using higher b-values, to diagnose 47 patients with cervical cancer. The study found that these models effectively distinguished cervical cancer from normal tissue, showing significantly lower mean ADC, D, and f (IVIM), as well as Dapp (ADC- DKI) and DDC (Distributed Diffusion Coefficient–SEM) values, while Kapp (DKI) and alpha (SEM) values were higher (*p* < 0.001). Combining modalities, particularly D, f, and D*, resulted in significantly improved outcomes, with logistic regression analysis indicating a larger (AUC; *p* < 0.05).

### Treatment

**Figure 1:**
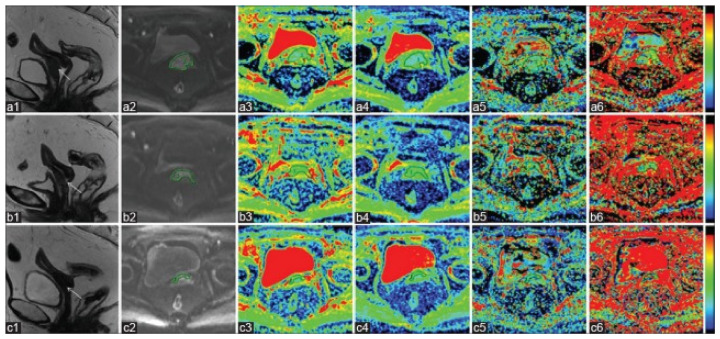
A 56-year-old woman with cervical squamous cell carcinoma (white arrow). Complete response after NACT. (**a1**–**a6**) Pre-NACT; (**b1**–**b6**) 3 weeks after the first NACT cycle; (**c1**–**c6**) 3 weeks after the second NACT cycle. (**a1**–**c1**) Sagittal T2-weighted images; (**a2**–**c2**) axial DWI with *b* = 800 s/mm^2^; (**a3**–**c3**) axial ADC maps; (**a4**–**c4**) D; (**a5**–**c5**) D*; (**a6**–**c6**) f values [28]. This figure was adapted from Wang et al. [28]. Originally published under the Creative Commons Attribution-Non-Commercial-ShareAlike 3.0 License. Permission for adaptation and reuse was also obtained from the original authors.

IVIM-DWI parameters—including D, D, f, and ADC—can be used to assess treatment response in cervical cancer (Figure 1). Figure 1 was adapted from Wang et al.’s paper [28]. IVIM has also been investigated for predicting lymph node metastasis [24] and evaluating the response to concurrent chemoradiation therapy (CCRT) [25].

A study examined the correlation between quantitative parameters derived from the IVIM model at baseline MRI and histological parameters, as well as the response to neoadjuvant chemotherapy (NACT) in patients with locally advanced cervical cancer (LACC). The authors noted that D values were significantly higher in patients with favorable responses and those with moderate to high levels of tumor-infiltrating lymphocytes, while fp values were found to be significantly greater in squamous cell tumors [29]. In another prospective study, Zhu et al. [30] reported that ADC and D values increased early during chemoradiotherapy, suggesting their utility as biomarkers of early treatment response.

Wang et al. [28] investigated the role of IVIM and ADC parameters for predicting and monitoring response to NACT in advanced cervical cancer. The study demonstrated that responders consistently showed higher ADC and D values than non-responders, whereas perfusion-related parameters (D* and f) showed limited predictive utility. These findings further support diffusion-related parameters as more reliable biomarkers of treatment response than perfusion metrics.

Kato et al. [31] conducted a prospective study assessing the utility of IVIM parameters for predicting early response to chemoradiotherapy in uterine cervical cancer. They reported that changes in IVIM-derived parameters (D*, f, D) and ADC between pre-treatment and post-treatment were significantly greater in the complete remission (CR) group than in the non-CR group.

In another prospective study, Li Zhu et al. conducted a prospective study to explore the application of IVIM parameters (ADC, D, D* and f) for predicting long-term prognosis in patients with advanced cervical cancers treated with CCRT. A total of 37 women with histologically confirmed advanced cervical cancers (FIGO IIA–IVB, all squamous cell carcinomas) were included and scanned at a 3T scanner [32]. Different time points were examined: within 2 weeks before CCRT and 2 and 4 weeks after the initiation of CCRT. Higher values predicted a good prognosis, while at the same time, the f value also performed well with an AUC of 0.820. A lower f value at Time Point 3 predicted a poor prognosis. A greater decrease in tumor size was observed in patients with favorable long-term outcomes, with a shrinkage rate in maximum diameter of ≥58.31% between Time Point 1 and Time Point 3 proving useful in predicting a positive prognosis (AUC = 0.751). However, the IVIM parameters provided earlier (at Time Point 2), and more accurate predictions (AUC of 0.857) were generally considered superior to tumor morphological information. The study also reported that all IVIM parameters, except for D*, demonstrated a significant positive relationship with various follow-up time points; however, the FIGO and age stage of the patients exhibited weak associations with these parameters.

Meng et al. [33] conducted a prospective study evaluating the diagnostic value of advanced MRI techniques—specifically IVIM and DKI—for differentiating cervical cancer from normal tissue, distinguishing between its pathological types (squamous cell carcinoma vs. adenocarcinoma), and correlating imaging parameters with the tumor’s degree of pathological differentiation. The study included 79 patients with cervical cancer (40 with adenocarcinoma, 39 with squamous cell carcinoma (SCC), and 30 HC). Various parameters were measured: ADC from DWI; D, D*, and f from IVIM; and MK (mean kurtosis) and MD (mean diffusivity) from DKI. They demonstrated that diffusion and kurtosis parameters correlated with histological subtype and tumor differentiation grade, with MK showing the strongest association with pathological differentiation.

A recent study [29] examined the relationship between baseline IVIM-derived quantitative metrics from MRI and both histological features and NACT outcomes in LACC. The researchers reported that elevated D values were significantly associated with favorable treatment response and with moderate-to-high levels of tumor-infiltrating lymphocytes. Additionally, the f was found to be significantly increased in tumors with squamous cell histology.

Bian et al. [34] conducted a prospective observational study evaluating the use of IVIM parameters for predicting treatment response to CCRT in cervical cancer. The study included 28 patients with biopsy-confirmed cervical cancer who underwent pelvic MRI at multiple time points, including 2 weeks before therapy, 7 days and 21 days after the therapy was initiated (during treatment), and 1 month after the treatment was completed. IVIM-derived parameters analyzed included ADCmin, ADCmean, ADCslow (D), ADCfast (D*), and Ffast (f). Participants were divided into two groups: a poor prognosis group (development of recurrence/metastases or with pathological tumor residue) and a good prognosis group (no recurrence or metastases during follow-up or without pathological tumor residue). The study found that pre-treatment ADC and diffusion-related IVIM parameters were associated with favorable treatment outcomes following chemoradiotherapy. ADCslow and Ffast demonstrated potential as early biomarkers of therapeutic response, supporting the role of IVIM-derived diffusion metrics in treatment monitoring.

**Table 1:** Summary of IVIM studies in cervical cancer.

Refs.	Type of Study/ # of Patients	# of b-Values	Parameters Used/ Outcome Measured	Main Findings
**Diagnosis & characterization**				
Wu et al. (2017) [21]	Prospective 141	**13** 0, 50, 100, 150, 200, 300, 500, 800, 1000, 1300, 1500, 1700, 2000 s/mm^2^	IVIM (D, D*, f, ADC) Differentiation of cervical cancer from normal tissue	D (b = 2000) showed best diagnostic performance (AUC = 0.923) for distinguishing cervical cancer from normal tissue.
Wang et al. (2021) [22]	Prospective 30	**6** 0, 30, 100, 200, 400, 1000 s/mm^2^	IVIM (D, f, D*) Comparison of fitting methods; cancer vs. normal tissue differentiation	D and f values were notably reduced in cancerous tissue compared to normal tissue, while no significant differences were observed for D*.
Becker et al. (2017) [23]	Prospective 19	**13** 0, 10, 20, 30, 40, 50, 75, 100, 150, 300, 500, 800, 1000 s/mm^2^	IVIM (D, D*, f) Feasibility assessment; histological subtype differentiation (SCC vs. adenocarcinoma)	Feasibility of parameter-free IVIM shown. Comparing squamous cell vs. adenocarcinoma, D did not differ significantly between the subtypes (0.89 [0.75–0.94] × 10^−3^ mm^2^/s vs. 0.90 [0.82–0.97] × 10^−3^ mm^2^/s, *p* = 0.27) whereas f (7.5% [7.0–9.0%] vs. 9.9% [9.0–11.4%] and D* (45.1 [25.1–60.4] × 10^−3^ mm^2^/s vs. 12.4 [10.5–21.2] × 10^−3^ mm^2^/s) differed significantly between the subtypes (*p* < 0.0).
Perucho et al. (2020) [24]	Retrospective 55	**13** 0, 10, 20, 30, 40, 50, 75, 100, 150, 300, 500, 800, 1000 s/mm^2^	IVIM (D, f, ADC) & DTV Prediction of pelvic lymph node metastasis	D best predicted pelvic LN involvement (AUC = 0.81). Combined D + DTV had 88% accuracy for LN metastasis.
Meng et al. (2023) [33]	Prospective 109: Group: 79 patients (SCC 39 & ACC 40) • HC Group: 30 subjects	**12** 0, 5, 10, 20, 40, 80, 160, 300, 600, 800, 1000, 2000 s/mm^2^	DWI (ADC), IVIM (D, f, D*), DKI (MD, MK) Cancer vs. normal differentiation; histological subtype; correlation with tumor grade	SCC showed lower D, f, D* vs. adenocarcinoma. MK correlated strongly with tumor grade. The ADC, D and MD values of cancer group were lower than those of HC group. All parameters showed significant differences (*p* < 0.05). D value demonstrated highest diagnostic performance (AUC = 0.991) with sensitivity and specificity is 100% and 97.4%, respectively. SCC vs. ACC: SCC showed lower D, D*, f, MD, ADC and higher MK values (*p* < 0.05). MK showed strongest positive correlation with differentiation grade (r = 0.796). D, MD and ADC showed negative correlation. MK was most effective for tumor grading. There was no statistical difference in f, D* and D between different menstrual states groups.
Song et al., 2022 [27]	Prospective 47	**8** 0, 30, 100, 200, 400, 1000,1500, and 2000 s/mm^2^	IVIM ADC, D, D*, f Diagnosis and staging	Monoexponential model, stretched exponential model and IVIM able to differentiate cervical cancer from normal tissue.
Lin et al., 2021 [26]	Prospective 90	**13** 0, 10, 20, 30, 40, 50, 75, 100, 150, 300, 500, 800, and 1000 s/mm^2^	DWI/IVIM ADC, D, D*, f Parametrial infiltration	Lower D and f in those with parametrial infiltration.
**Treatment response & monitoring**				
Zhang et al. [25]	Prospective 84	**12** 0, 10, 25, 50, 75, 100, 150, 200, 400, 800, 1000, 1500 s/mm^2^	IVIM (D, D*, f), SEM (DDC, α) Prediction of response to CCRT	SEM’s Distributed Diffusion Coefficient (DDC) best predicted response to CCRT (AUC = 0.948). Perfusion parameters (D*, f) showed no significant difference between responders/non-responders. The DDC, D, and ADC values were lower in responders than in non-responders group (*p* = 0.01, 0.02, 0.03).
Zhu et al. (2016) [30]	Prospective 21	**14** 0, 10, 20, 30, 40, 50, 100, 150, 200, 350, 500, 650, 800, 1000 s/mm^2^	IVIM (ADC, D, f, D*) Early treatment response monitoring during CCRT	ADC & D increased during CCRT; f & D* rose initially (2 wks) then declined (4 wks), indicating early perfusion changes.
Wang et al. (2016) [28]	Prospective 42	**9** 0, 50, 100, 150, 200, 300, 400, 600, 800 s/mm^2^	IVIM (ADC, D, D*, f) Prediction of response to NACT	Pre-treatment D & ADC higher in responders to NACT. No significant difference in D* & f between groups. Analysis of the ROC curves indicated that an ADC value threshold < 1.11 × 10^−3^ mm^2^/s and D threshold < 0.93 × 10^−3^ mm^2^/s could distinguish responders from non-responders at pre-NACT time point, yielding AUC of which were 0.806 and 0.771 respectively. The AUC values for D and ADC at these three time points did not exhibit significant differences (*p*-values of 0.641, 0.512, and 0.547, respectively).
Kato et al. (2019) [31]	Prospective 17	**6** 0, 50, 100, 200, 400, 800 s/mm^2^	IVIM (ADC, D, D*, f) Early response prediction to chemoradiotherapy	Early % changes in ADC, D, D*, and f at 20 Gy predicted complete remission. Only Δ%ADC remained significant at 40 Gy.
Bian et al. (2019) [34]	Prospective 28	**5** 0, 50, 200, 450, 850 s/mm^2^	IVIM (ADC min, ADC mean, D, D*, f) Treatment response prediction and prognosis assessment	Pre-treatment ADC min & D higher in good prognosis group. f change rate at day 7 was higher in responders. ADC changes during treatment and pre-treatment correlate with response.
Zhu et al. (2017) [32]	Prospective 37	**9** 0, 25, 50, 75, 100, 150, 200, 500, and 800 s/mm^2^	IVIM (ADC, D, f, D*) Long-term prognosis prediction after CCRT	ADC at 4 weeks after initiation of CCRT showed the highest prognostic performance (AUC = 0.857). Higher ADC and D values were associated with favorable treatment outcomes, whereas lower f values were associated with poorer prognosis. Diffusion-related parameters demonstrated greater predictive value than perfusion-related metrics

Although most IVIM studies report promising diagnostic and predictive performance, substantial methodological heterogeneity persists across the literature. Major differences in b-value selection, fitting algorithms, magnetic field strength, acquisition protocols, and ROI placement methods significantly affect the reproducibility and comparability of IVIM-derived parameters across studies [13,14].

Among IVIM metrics, diffusion-related parameters such as ADC and D show the most consistent associations with tumor cellularity, histological grade, and treatment response. In contrast, perfusion-related parameters (D* and f) show conflicting findings and lower reproducibility, likely due to their sensitivity to noise and dependence on low-b-value acquisition [14,19,26]. This inconsistency limits their immediate clinical applicability despite their theoretical biological relevance.

Importantly, the strongest evidence for IVIM appears in treatment response assessment, where early increases in ADC and D values consistently precede morphological tumor regression during chemoradiotherapy or NACT [20,22,24]. Specifically, compared to non-responders, ADC and D had significantly higher values than responders. These findings suggest that advanced diffusion MRI may provide clinically meaningful functional biomarkers that identify treatment responders earlier than conventional anatomical MRI. However, despite encouraging findings, most available studies remain limited by small sample sizes, single-center designs, and a lack of external validation, thereby restricting translation into standardized clinical protocols.

## 5. Application of DTI in Cervical Cancer

Only a few studies met the inclusion criteria, with a combined sample size of approximately 100 patients (Table 2). Therefore, any conclusions regarding its diagnostic or prognostic value should be interpreted with caution.

Yamada et al. [35] conducted a prospective study correlating DTI parameters with histopathologic findings to non-invasively assess several prognostic factors, including lymph node metastasis, histologic grade and tumor invasion depth. The analysis focused on three key DTI parameters calculated on a voxel-by-voxel basis: FA, MD, and AD. The study found that DTI parameters are inversely correlated with tumor aggressiveness. The mean FA value decreased progressively as the tumor grade worsened: Grade 2 (moderately differentiated) exhibited a mean FA of 0.326 ± 0.010, while Grade 3 (poorly differentiated) showed a mean FA of 0.260 ± 0.054. Tumor tissue consistently showed lower FA, MD, and AD values than the normal uterine cervical wall layers. Furthermore, DTI parameters successfully differentiated between metastatic and nonmetastatic pelvic lymph nodes. Metastatic lymph nodes showed significantly lower FA, MD, and AD values compared to nonmetastatic nodes. The parameters demonstrated high diagnostic utility, with the optimal FA cut-off value of ≤0.359 yielding an AUC of 0.958 for predicting metastasis, making DTI a promising non-invasive tool for preoperative assessment and staging. The study concluded that DTI parameters correlated with tumor grade and lymph node metastasis, suggesting potential value for assessing tumor aggressiveness. Lin et al. [26] conducted a prospective study to determine the diagnostic value of combined IVIM and DTI for predicting parametrial infiltration in cervical cancer. DTI parameters revealed a statistically significant difference in FA values between groups (*p* = 0.003). Specifically, the parametrial infiltration-positive group had a significantly lower FA (0.073 ± 0.002) compared to the parametrial infiltration-negative group (0.085 ± 0.003). When DTI-derived FA was combined with the D from IVIM analysis, the diagnostic performance significantly improved, yielding an AUC of 0.931. IVIM and DTI metrics assessed in parametrial infiltration were significantly lower than those in non-infiltrated tissue. Specifically, D-IVIM values decreased (0.63 vs. 0.77), and FA values on DTI were reduced (0.07 vs. 0.09). Combining IVIM and DTI provided the highest diagnostic performance, with improved ROC-AUC values for D (from 0.80 to 0.93) and for FA (from 0.73 to 0.93).

Di Paola et al. [36] prospectively evaluated the efficacy of DTI of the lumbosacral plexus for detecting PMI in 27 women with histopathologically confirmed cervical cancer using 1.5 T. They found that the mean FA value was significantly higher in invaded parametria (0.321 ± 0.036) compared to non-invaded parametria (0.292 ± 0.02, *p* = 0.01). The best cut-off value of FA for diagnosing PMI was >0.3099. Using this cut-off, DTI demonstrated an accuracy of 66% (95% CI: 54.62–73.91). Inter-observer agreement for FA values was high (ICC values ranging from 0.88 to 0.92). DTI shows preliminary potential, but current evidence is insufficient to support its routine clinical use.

Compared with IVIM, the evidence base supporting DTI in cervical cancer remains considerably smaller and less mature. Most studies included limited patient cohorts and focused primarily on tumor grading and PMI. Nevertheless, the available evidence suggests that FA may provide useful information regarding stromal invasion and tissue microstructural disruption [26,35,36]. The combination of DTI-derived FA with IVIM-derived diffusion parameters appears particularly promising for improving detection of PMI, a critical determinant of FIGO staging and treatment selection. However, contradictory findings regarding FA behavior in invaded parametrial tissue indicate that the biological interpretation of DTI parameters in cervical cancer remains incompletely understood [26]. Furthermore, the limited number of studies, variability in acquisition techniques, and absence of standardized post-processing methods currently prevent definitive conclusions regarding the routine clinical role of DTI in cervical cancer imaging. Consequently, DTI should presently be considered an investigational adjunct rather than a validated clinical biomarker.

### Combined DWI Techniques with Radiomics

Radiomics converts medical imaging data into high-dimensional quantitative features that reflect the underlying tumor biology. This approach facilitates the quantitative and high-throughput extraction, as well as the thorough analysis, of the biological characteristics and heterogeneity of tumors [37,38]. In cervical cancer research, radiomics studies have primarily concentrated on predicting lymph node metastasis [39,40], while other investigations have explored lymphovascular space invasion [41] and prognostic outcomes [40,42].

Although MRI is the primary imaging modality, quantitative radiomic analysis using IVIM and DTI in cervical cancer remains limited. These techniques have been applied for preoperative staging and evaluating treatment efficacy in patients with cervical cancer. Thapa et al. [43] suggested that the histogram feature representing the 75th percentile, extracted from the f, D*, D and ADC maps, can effectively differentiate between early-stage and LACC. Another retrospective study evaluated the impact of intra- and inter-observer variability on the stability of IVIM parameters (f, D*, D, and ADC) using radiomics features in 25 patients with cervical cancer. A total of 105 radiomic features were ultimately extracted from IVIM-derived maps. The study reported that radiomic features extracted from the ADC, D, and D* maps exhibited greater reproducibility compared to those derived from the f maps. In the intra-observer analysis, the reliability of reproducible features for the D*, ADC, D, and f maps ranged from 95% to 100%, 89% to 93%, 86% to 97%, and 54% to 62%, respectively. Conversely, the reproducibility rates in the inter-observer analysis were 90% to 94% for the D* map, 70% to 85% for the ADC map, 78% to 81% for the D map, and 41% to 93% for the f map. Overall, the ADC, D, and D* maps demonstrated strong performance in both intra- and inter-observer analyses, whereas the f map showed suboptimal performance in both evaluations [44]. A retrospective analysis of 163 patients (divided into training and test groups) who underwent IVIM-DWI before CCRT classified patients as resistant or sensitive based on treatment efficacy at 6 months after CCRT. This study aimed to assess the value and feasibility of IVIM parameters (ADC, D, D*, and f values) and MRI-based radiomics in conjunction with clinical prognostic factors for predicting the sensitivity of CCRT in LACC. All parameters except D demonstrated independent diagnostic significance in multivariate logistic regression analysis. The resulting predictive model achieved AUC values of 0.987 and 0.984 for the training and test groups, respectively. The authors concluded that a prediction model combining IVIM parameters (D and f values), clinical stage, and radiomic features (SizeZoneNonUniformity, Minimum, and InverseVariance) achieved high predictive performance for CCRT sensitivity in LACC [45].

Another retrospective study [46] predicted early treatment response to CCRT for cervical cancer patients by integrating texture analysis with IVIM parameters and creating a nomogram for assessing the risk of residual tumor presence. The study included 93 cervical cancer patients. Multivariate analysis of texture features revealed that GLSZM-ZE, GLRLM-LRE and GLCM-correlation were independent predictors (OR  =  23.738, 9.774 and 43.789, respectively; *p* < 0.05), whereas IVIM parameters showed that f and D were independent predictors (OR  =  0.889 and 4.029, respectively; *p* < 0.05). Moreover, the combination of texture features and IVIM parameters showed the highest predictive performance (AUC  =  0.975) for distinguishing partial from complete responses. The developed nomogram demonstrated robust predictive performance and stability in identifying patients with high-risk residual tumors, yielding a Concordance index (C-index) of 0.953.

A recent prospective study [47] investigated the critical challenge of long acquisition times in multi-b-value DWI for cervical cancer radiomics by analyzing the impact of simultaneous multislice acceleration (SMS) on feature stability. The aim was twofold: first, to systematically determine how SMS acceleration factors (AFs 1, 2, and 3) and tumor segmentation dimensions (two-dimensional (2D) vs. three-dimensional (3D)) influence the stability of radiomics features derived from IVIM (D, D*, f) and DKI parametric maps. Second, the study aimed to identify a subset of highly stable radiomics features that can characterize the clinical stages of cervical cancer (low-stage vs. high-stage). The study included 40 patients with pathologically confirmed cervical cancer, all of whom underwent multi-b-value DWI before treatment. Patients were stratified by clinical severity according to the FIGO staging system, ranging from stage IB to IV. The study reported that feature stability decreased with higher acceleration factors. Among the evaluated features, 12.7% of the three-dimensional (3D) and 9.1% of the two-dimensional (2D) radiomic features were stable, defined as a concordance correlation coefficient greater than 0.9 and COV less than or equal to 0.1. The D* and f maps showed the lowest stability, while ADC maps exhibited the highest stability, with 3D features more stable than their 2D counterparts. Ultimately, five stable 2D features and 25 stable 3D features were identified that effectively distinguished between lower and higher stages, with AUC ranging from 0.66 to 0.83. The extremely high AUC values reported in some radiomics studies should be interpreted with caution, given the risk of overfitting, lack of external validation, and the high dimensionality of radiomic features relative to sample size.

## 6. Discussion

The integration of advanced DWI sequences, specifically IVIM and DTI, has fundamentally enhanced the ability to characterize cervical cancer beyond mere morphology. While conventional MRI provides a necessary anatomical framework for staging, it is largely limited to assessing tumor size, signal intensity, and morphology. In contrast, the quantification of water molecular kinetics and microvascular perfusion offers a non-invasive window into the tumor’s pathophysiological microenvironment.

As summarized in Table 1 and Table 2, diffusion-related MRI parameters demonstrated greater consistency and reproducibility than perfusion-related metrics across studies, highlighting the current methodological limitations affecting clinical translation of advanced diffusion MRI techniques in cervical cancer. Despite promising preliminary findings, the evidence supporting DTI in cervical cancer remains limited by small sample sizes, methodological variability, and insufficient multicenter validation.

The literature consistently demonstrates that malignant tissues exhibit significantly lower ADC and D values compared to healthy stroma or benign lesions. These metrics serve as direct indicators of high cellular density and restricted extracellular space, with the D parameter demonstrating exceptional diagnostic performance, reaching AUC values as high as 0.991 in some cohorts.

Conventional T2-weighted MRI remains the cornerstone of local staging of cervical cancer, providing exquisite anatomical detail. Nevertheless, its limitations in characterizing intrinsic tumor heterogeneity, cellular density, and microvascular environments are well-recognized. Advanced quantitative sequences fill this critical gap. The consistent finding across studies that the ADC and the D from IVIM are significantly lower in malignant compared to benign or normal tissue underscores their validity as biomarkers of increased cellularity and restricted extracellular space [5,8,21]. This functional data provides a direct, non-invasive correlate of histopathological tumor grade, with poorly differentiated neoplasms typically demonstrating the most restricted diffusion [20]. Furthermore, the ability of these parameters to differentiate histological subtypes, such as the generally lower D and f values in squamous cell carcinoma versus adenocarcinoma, hints at a future where imaging can inform biological subtyping [23,32].

To improve local staging of cervical cancer, the combination of DTI-derived FA and IVIM-derived D has demonstrated superior diagnostic performance over anatomical imaging alone for detecting PMI (AUC 0.931) [26]. This is of paramount importance in distinguishing surgical candidates from those requiring primary chemoradiation.

The most compelling application is monitoring response to CCRT or NACT. A reproducible early increase in ADC and D values, observable within 2–4 weeks of treatment initiation, reflects therapy-induced cellular necrosis and loss of membrane integrity [27]. This ‘functional response’ often precedes tumor shrinkage, offering a critical window for early identification of non-responders. The dynamic behavior of perfusion parameters (f and D*), usually showing an initial rise followed by a decline, may provide additional insight into vascular changes during treatment [27,30].

Despite these encouraging results, the literature is marked by inconsistencies stemming from a lack of standardization, which impede clinical translation. There is no consensus on optimal acquisition parameters. As demonstrated in Table 1, there is no consensus regarding the optimal number and distribution of b-values. While studies like Bain et al. [34] utilize only five b-values for rapid clinical acquisition, more comprehensive protocols such as those by Zhu et al. [30] employ up to 14 b-values to ensure a more robust characterization of the biexponential IVIM decay curve, particularly in the low b-value range (<200 s/mm^2^), which is critical for accurate perfusion (D*) estimation. These disparate sets of b-values and different fitting algorithms for IVIM parameter calculation can directly affect the absolute values and reproducibility of derived metrics [21,22]. This variability likely contributes to the contradictory diagnostic utility reported for the noise-sensitive perfusion parameters D* and f across studies [22,27]. While ADC and D correlate with cellularity, the precise histopathological correlates of D* and f in the complex tumor microenvironment of cervical cancer remain unclear [8,33]. Similarly, the biological basis for altered FA in tumor stroma requires further correlation with histologic markers of invasion [34]. Perfusion parameters exhibit lower inter-reader agreement than the D and ADC [23]. Their stability is further compromised by accelerated acquisition techniques, such as SMS imaging, which are essential for clinical feasibility but have been shown to degrade feature stability [47].

Advanced diffusion MRI (IVIM and DTI) parameters may also provide clinically relevant information for cervical cancer staging. Several studies demonstrated associations between lower diffusion values, altered perfusion characteristics, and more advanced disease, particularly in cases with PMI [26,35,36]. For example, one study demonstrated that FA, MD, and AD were significantly associated with lymph node metastasis, histologic grade and tumor invasion depth. These findings suggest that DTI may complement conventional MRI by providing additional functional information relevant to disease extent and tumor aggressiveness. Additionally, lower ADC and D values have been associated with higher tumor cellularity and more advanced disease. At the same time, combined IVIM-DTI approaches demonstrated promising performance for detecting PMI, a key determinant of FIGO stage. These findings suggest that IVIM and DTI may complement conventional MRI by providing additional functional information relevant to FIGO staging and treatment planning. Integration of these imaging findings with clinical examination, operative assessment, and histopathological analysis of resected specimens may further improve staging accuracy, treatment planning, prognostic evaluation, and post-treatment monitoring. However, larger prospective studies integrating imaging, clinical, operative, and pathological findings are still required before routine clinical implementation can be established.

Accurate staging remains the primary factor in determining whether a patient is a candidate for surgery or primary radiation. DTI is particularly sensitive to the disruption of the organized collagenous structures in the parametrium. When DTI-derived FA is combined with IVIM-derived diffusion metrics (D), the diagnostic performance for detecting parametrial infiltration significantly improves, yielding an AUC of 0.931 [26]. Similarly, DTI parameters have demonstrated high diagnostic utility in differentiating metastatic from non-metastatic pelvic lymph nodes, with FA values providing an AUC of 0.958 for predicting metastasis. This preliminary evidence suggests that DTI parameters may offer diagnostic utility, though this is based on only limited studies and requires confirmation.

In several malignancies, including prostate, breast, and liver cancers, IVIM and DTI diffusion MRI techniques have already influenced imaging protocols by improving lesion characterization, treatment response monitoring, and risk stratification [9,10,11,14,15]. For example, multiparametric MRI incorporating diffusion imaging is now integrated into standardized prostate cancer diagnostic pathways and treatment planning. In breast and hepatic imaging, IVIM-derived biomarkers have demonstrated clinical utility in distinguishing malignant from benign lesions and evaluating treatment response. In contrast, despite promising preliminary evidence, cervical cancer imaging protocols remain largely dependent on conventional anatomical MRI sequences. This discrepancy likely reflects the substantial methodological heterogeneity across cervical cancer studies, limited multicenter validation, lack of standardized acquisition parameters, and insufficient evidence of reproducible incremental clinical benefit over established MRI protocols. Consequently, although these techniques show potential for improving functional characterization and early treatment monitoring in cervical cancer, current evidence remains insufficient to support routine incorporation into standardized diagnostic pathways.

Despite these advancements, the transition from research to routine clinical practice faces several challenges. The limitations of IVIM diagnostics arise from the lack of standardized fitting procedures for the IVIM biexponential function and the scarcity of studies that validate the reproducibility of this method. Quality control measures involve comparing the diagnosis of cervical cancer through IVIM parameters with histological findings or traditional clinical assessments. Additionally, while diffusion parameters like D are generally stable, perfusion-related metrics like D* remain sensitive to signal noise and show lower inter-reader agreement. As the field moves forward, the use of acceleration techniques such as SMS imaging will be essential to reduce scan times, though feature stability must be carefully monitored as it tends to diminish with higher acceleration factors.

A key observation across the reviewed studies is the differential reliability of diffusion-related versus perfusion-related parameters. Diffusion parameters, such as ADC and D, consistently demonstrate associations with tumor cellularity and treatment response, reflecting their direct relationship with tissue microstructure. In contrast, perfusion-related parameters (D* and f) exhibit substantial variability and inconsistent findings across studies. This inconsistency is likely attributable to several technical factors, including high sensitivity to noise, dependence on low b-value acquisition, and variability in fitting algorithms used for parameter estimation. These limitations reduce the robustness and reproducibility of perfusion parameters, thereby limiting their current clinical applicability.

This review provides a novel integrative perspective by distinguishing the differential clinical reliability of diffusion versus perfusion parameters and highlighting the technical factors underlying inconsistent findings. Furthermore, it emphasizes the emerging role of combined models and radiomics, which may represent the next phase in quantitative cervical cancer imaging.

### Critical Limitations and Technical Challenges

Despite the performance advantages of advanced models, the standard ADC remains the most stable metric in the presence of technical variations. However, a primary challenge in implementing IVIM and DTI for routine MRI is the lack of standardization in both image acquisition and analysis, resulting in considerable variability in the calculated parameters across different studies. Consequently, further refinement of this technique is needed regarding its application, analysis, and acquisition. Furthermore, the lack of standardized ‘b-values’ across studies makes D and FA values harder to generalize compared to the widely accepted ADC. This review was conducted by a single author, which may introduce selection and interpretation bias. Ideally, study selection and data extraction should be performed by multiple independent reviewers. Furthermore, formal methodological quality assessment and risk-of-bias evaluation of the included studies were not performed because this work was designed as a narrative rather than a systematic review.

Despite encouraging findings, several important limitations currently restrict the clinical translation of these techniques in cervical cancer. The available literature is characterized by substantial methodological heterogeneity, including differences in b-value selection, acquisition protocols, magnetic field strength, ROI placement methods, and fitting algorithms. Most studies were single-center investigations with relatively small sample sizes and limited external validation. Furthermore, the reproducibility of perfusion-related IVIM parameters remains inconsistent because of their sensitivity to noise and dependence on low b-value acquisition. These limitations complicate direct comparison across studies and currently prevent standardization of these techniques for routine clinical implementation.

To facilitate clinical translation, future studies should adopt minimum reporting standards, including: (1) detailed acquisition parameters (b-value distribution, field strength, acceleration factors); (2) explicit description of fitting algorithms; (3) inter-reader reproducibility metrics; and (4) external validation where feasible. The use of abbreviated protocols with 6–8 carefully selected b-values may offer a pragmatic balance between acquisition time and parameter robustness. Future research should investigate the integration of these advanced techniques with conventional MRI, PET-CT, radiomics, and clinical examination findings to improve diagnostic accuracy and therapeutic stratification. Multimodal imaging approaches may provide a more comprehensive assessment of tumor biology and treatment response than single-technique evaluation alone.

## 7. Conclusions

Advanced IVIM and DTI-DWI techniques, particularly IVIM, show promise for improving tumor characterization and treatment monitoring in cervical cancer. DTI should currently be considered an investigational adjunct rather than an established clinical biomarker in cervical cancer imaging. However, unlike breast and prostate imaging, where these techniques have become integrated into multiparametric diagnostic frameworks, cervical cancer applications remain investigational because of methodological inconsistencies and insufficient prospective validation. The differential reliability of diffusion versus perfusion parameters underscores the need for standardized acquisition and analysis protocols. Establishing standardized acquisition protocols and reproducible multicenter evidence will be essential before these techniques can be incorporated into routine cervical cancer imaging algorithms. Future studies should investigate the integration of radiomics and combined multiparametric imaging models to further enhance diagnostic performance and prognostic assessment in cervical cancer.

## Figures and Tables

**Table 2:** Summary of DTI studies in cervical cancer.

Refs.	Type of Study/# of Patients	Parameters Used	Main Findings
Yamada et al. (2020) [35]	Prospective 15	DTI (FA, MD, AD) Correlation with tumor grade; differentiation of metastatic lymph nodes	DTI parameters (FA, MD, and AD) showed significantly inverse correlations with the histologic grades of cervical cancer. FA inversely correlated with tumor grade (AUC = 0.893). FA ≤ 0.315 differentiated high- vs. low-grade tumors. Lower FA, MD, and AD in metastatic LNs (AUC = 0.958).
Lin et al. (2021) [26]	Prospective 90 (65 for DTI)	DTI (FA), IVIM (D) Detection of parametrial infiltration	FA lower in PMI + group. Combined FA (DTI) + D (IVIM) gave best AUC (0.931) for PMI detection.
Di Paola et al. (2022) [36]	Prospective 27	DTI (FA) Detection of PMI	Higher FA in invaded parametria (0.321 ± 0.036) vs. non-invaded (0.292 ± 0.02). FA > 0.3099 predicted PMI with 66% accuracy.

## Data Availability

No new data were created or analyzed in this study.
